# Population profile and residential environment of an urban poor community in Dhaka, Bangladesh

**DOI:** 10.1186/s12199-017-0610-2

**Published:** 2017-03-14

**Authors:** Md. Khalequzzaman, Chifa Chiang, Bilqis Amin Hoque, Sohel Reza Choudhury, Saika Nizam, Hiroshi Yatsuya, Akiko Matsuyama, Yoshihisa Hirakawa, Syed Shariful Islam, Hiroyasu Iso, Atsuko Aoyama

**Affiliations:** 1 Department of Public Health and Informatics, Bangabandhu Sheikh Mujib Medical University, Dhaka, Bangladesh; 2 Department of Public Health and Health Systems, Nagoya University School of Medicine, Nagoya, Japan; 3 Environment and Population Research Center, Dhaka, Bangladesh; 4 Department of Epidemiology and Research, National Heart Foundation Hospital and Research Institute, Dhaka, Bangladesh; 5 Department of Public Health, Fujita Health University School of Medicine, Toyoake, Aichi Japan; 6 Nagasaki University School of Tropical Medicine and Global Health, Nagasaki, Japan; 7 Public Health Graduate School of Medicine, Osaka University, Suita, Osaka Japan

**Keywords:** The urban poor, Bangladesh, Baseline population survey, Residential environment, Non-communicable diseases

## Abstract

**Objectives:**

A population survey was conducted in an urban shantytown in Bangladesh, as a baseline study of future epidemiological studies. This paper aims to describe the findings of the study, including the population profile and residential environment of the urban poor.

**Methods:**

We conducted a complete count household survey in an urban poor community in Dhaka. Using a brief structured questionnaire in Bengali language, trained interviewers visited each household and asked questions such as: duration of residence; ownership of house, toilet and kitchen; water supply; number of family members; age, sex, education, occupation, tobacco use, and history of diseases of each family member.

**Results:**

We found that there were 8604 households and 34,170 people in the community. Average number of household members was 4.0. Most people had access to safe water, but only 16% lived in the house with a toilet. Based on the proxy indicators of household wealth levels, we identified that about 39% were relatively well-off, while the rest were very poor. Tobacco use was prevalent in men regardless of age and in women aged over 35 years. Prevalence of self-reported hypertension and diabetes was slightly higher in women than in men, although over 70% of the respondents didn’t know if they had such diseases. Incidences of diarrhea in the last one month were relatively low.

**Conclusions:**

The study showed population profile and sanitation environment in an urban poor community by a complete count survey. We expect the study to serve as a baseline for future epidemiological studies.

## Introduction

Bangladesh is a lower-middle income country in South Asia, with over 160 million population in 2015 [1]. The population of the metropolitan area of the capital city Dhaka was around 17 million. Urban population in Bangladesh is rapidly increasing, as indicated by 3.4% annual urban population growth in comparison with 1.2% population growth in the whole nation in 2015.

Infectious diseases are still prevalent in Bangladesh, mostly due to poor sanitation environment. In addition, the burden of non-communicable diseases (NCDs) is also increasing: age-standardized mortality rates of all NCDs, cardiovascular diseases, and diabetes were 548.9, 166.2, and 29.8 per 100,000 population in 2012, respectively [2]. Previous surveys on NCD risk factors in Bangladesh showed that the prevalence was higher in urban areas than in rural areas [3–7], and NCDs were prevalent even among the poor in a rural area [8].

However, little has been known about the situation of NCD risk factors among the poor living in urban shantytowns, where lifestyles are changing rapidly. In addition, low birth weight and childhood malnutrition might be more prevalent among the poor, which may reportedly increase the risks of cardiovascular diseases and diabetes in adulthood [9, 10]. Therefore, the urban poor could be considered as high risk population, but proper NCD control measures are difficult to be taken. One of the reasons of this difficulty is the unknown situation of the population group.

Since no reliable recent census are available in shantytowns where people move in and out without registration, it is difficult to conduct epidemiological surveys based on representative sampling methods. Therefore, we conducted a population survey targeting all households in a shantytown as a baseline of future epidemiological surveys. In addition to demographic data, we also collected some information related to residential environment and awareness of NCDs, as they may help us make a plan of future studies.

This paper aims to describe the findings of the baseline study, including population profile, residential sanitation environment, and prevalence of self-reported NCD risk factors among the urban poor.

## Methods

### Study site and study population

We conducted a census-like baseline population study in Bauniabadh, an urban poor community in Mirpur ward, Dhaka city, Bangladesh [11–13]. The community was originally established by the government in 1972 as a settlement for the poor. A same size land plot (about 8.9 m^2^) was allocated to each household at an affordable price. Since then, many residents moved in or out without registration, and the community expanded irregularly with sprawling shantytowns in and outside the original boundary. Before this study, it was estimated that there were about 15,000 households (about 50,000 population) within the original boundary and about 5000 households in the areas adjacent to the original boundary.

**Fig. 1:**
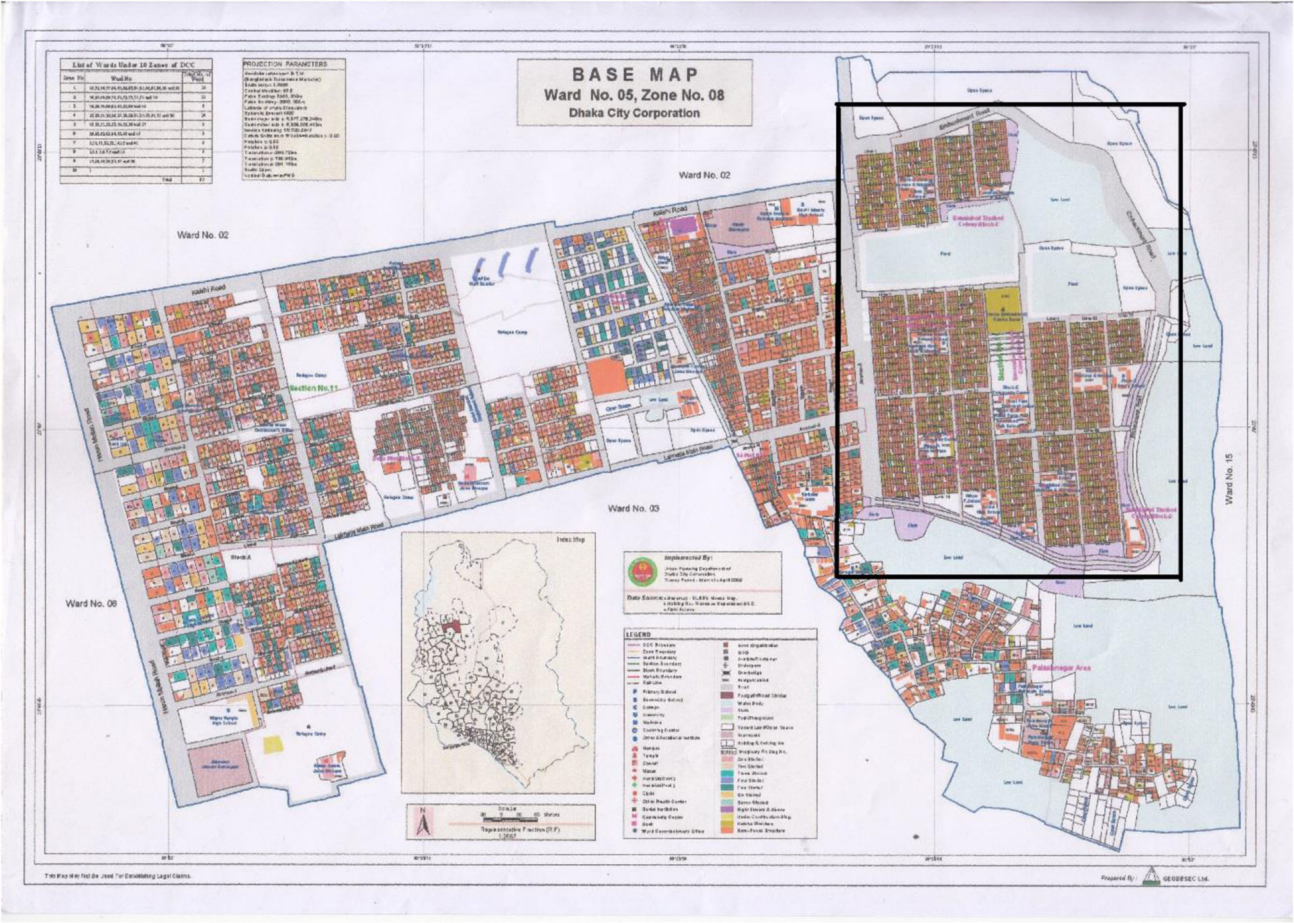
Map of the target community. The framed area is the target community, Bauniabadh. (Source: Urban Planning Department, Dhaka North City Corporation, Dhaka, Bangladesh)

We targeted all households within the original boundary but excluded the surrounding shantytowns, considering the feasibility of the study (Fig. 1). The original area was consisted of 5 blocks; each block consisted of 22 lanes (except a block consisted of 24 lanes); each lane consisted of 24 plots. Although each household allocated each one plot originally, some plots were combined to build multistoried buildings and it was not known how many households shared the building.

### Staff training and community mobilization

We recruited 14 field interviewers, who lived in the same community and completed secondary level education, and trained them for interview skills. Two field supervisors were assigned for managing field activities and controlling data quality.

Mobilizing the community and encouraging people to participate in the survey, meetings with community leaders and other representatives were held in the community several times before and during the survey period. Community leaders were actively involved in motivating people to participate.

### Data collection

We conducted the baseline population survey from August to December, 2014. We targeted all households living in the original 5 blocks of Bauniabadh, including tenants in rented and sublet houses, and people living apart from their original households.

We prepared a brief structured questionnaire in Bengali language. Each household was asked following questions: type of housing structure, owner or tenant, duration of residence, ownership of toilet and kitchen, source of water supply, satisfaction with water quality, and number of household members. Questions about each household member were as follows: age, relation to the household head, sex, religion, education, occupation, marital status, tobacco use, and history of diseases (hypertension, diabetes, heart diseases, stroke, and diarrhea in the last 1 month). Unknown categories for the history indicated that the respondent did not know if they had hypertension, diabetes, heart disease, or stroke. The questionnaire was revised several times until all the interviewers were confirmed to be able to confidently complete the interview.

The trained interviewers visited each household of the community and conducted face to face interviews in Bengali language to the household head, or a household member who could give information of all household members if the household head was unavailable. The interviewer also observed housing structure, kitchen, toilet, and water supply of the household. The supervisors monitored the interviewers intermittently in the field to make sure the questions were properly administered.

To avoid overlapping or skipping of households in the crowded shantytown, we used the following procedures. We first selected one lane of each block. The interview started from the first household of the north-west corner of the selected lane. Before starting the interview, the interviewer got permission of the resident and marked an identification code number on the edge of the main entrance door with a permanent marker, and noted the same code number in the questionnaire. After finishing the interview, the interviewer selected the next household on the right side. Once the interviews of all households in the right side of the lane were completed, the interviewer turned around and started the interviews of the households on the other side of the lane. After finishing the lane, the interviewer reported to the supervisor, and the supervisor confirmed all households in the lane had been covered. It took at least 4 days to complete interviews of all households in one lane.

In case of multistoried building, the interview started the household on the right side of the stair at the ground floor. The interviewer moved to upwards visiting household at each floor and reached the household at the top floor. Then the interviewer turned around and interviewed households on the other side of the stair from the top floor to the ground floor.

If a sublet family lived together in the same house, the household interview was conducted to the sublet family separate from the host family. In case the house was locked or no one was available for the interview, the interviewer marked the code number on the door and noted the number on the questionnaire. The interviewer returned the household when the household member was available, and conducted the interview using the code number.

### Data analysis

The household residents’ names are separated from the original sheets, which are coded with serial numbers. The anonymized data were inputted into a programmed data entry template and subjected to statistical analyses. All of the statistical analyses were performed using the statistical software, IBM SPSS Statistics for Windows, Version 23.0 (IBM Corp, Armonk, NY, USA). Differences between all men and all women were tested by using age-adjusted binary and multinominal logistic regression models for dichotomous and trichotomous variables, respectively.

### Ethical considerations

This study was reviewed and approved by the Bioethics Review Committee of Nagoya University School of Medicine, Japan (approval no. 2014-0021). Institutional Review Boards of Bangabandhu Sheikh Mujib Medical University and National Heart Foundation Hospital and Research Institute, Bangladesh, approved the study as well. Written informed consents were obtained from all participants after adequate explanations of the study.

## Results

We found that there were 8604 households in the 5 blocks of the original Bauniabadh and 34,170 people (men 17,041; women 17,129) resided. The household number and population were much less than we estimated before the study.

Table 1 shows demographic and residential characteristics of the study population (*n* = 34,093 excluding 77 without age information). Average number of household members was 4.0, suggesting that most households in the urban poor community were nuclear families and average fertility was similar to the national total fertility rate (2.2 in 2013) [2]. About 25% of adults lived in the community for 5 years or less, indicating frequent migration of the urban poor. Only 0.4% lived there since the community was established, and 35% were born in the community.

**Table 1:** Demographic and residential characteristics of the study population

Total population	34 093
Mean number of household members	4.0
Native-born (%)	35.1
Dwelling since 1972 (%)	0.4
New comers in adults aged ≥18 years^a^ (%)	24.7
Years of formal education in adults aged ≥18 years (%)	
None	32.0
1–4	16.5
5–7	23.7
8–9	15.5
≥ 10	12.3
Occupation in adults aged ≥18 years (%)	
Employed	25.9
Self-employed	15.5
Day labor	19.0
Homemaker	27.5
Others	12.1
Type of housing structure (%)	
Concrete roofs/brick walls/concrete floors (*pucca*)	38.5
Tin roofs/brick walls/mud or wooden floors (*semi-pucca*)	31.4
Tin roofs/thatch or bamboo walls/mud or wooden floors (*kutcha*)	30.1
House ownership (%)	
Owned	41.5
Rented	57.4
Others	1.0
Self-owned toilet (%)	15.6
Self-owned kitchen (%)	18.7
Source of drinking water (%)	
Piped water	99.0
Shallow tube well	1.0
Satisfaction with water quality (%)	62.5

^a^Those who had lived in the community for ≤5 years

**Fig. 2:**
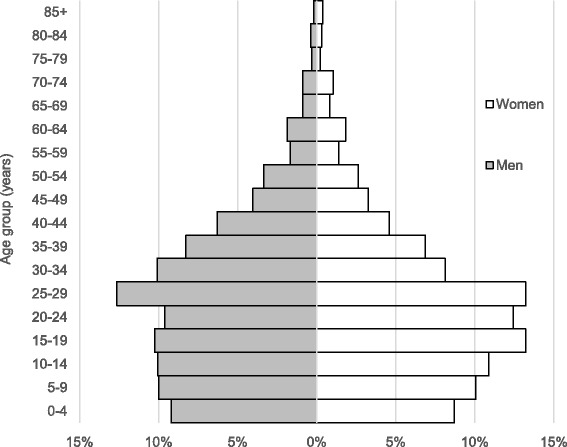
Population by sex and age groups

Figure 2 shows population by sex and age groups, forming a constrictive type of population pyramid. While the pyramid indicated that birth rates were decreasing, the population was still young, as about 80% were less than 40 years of age.

**Fig. 3:**
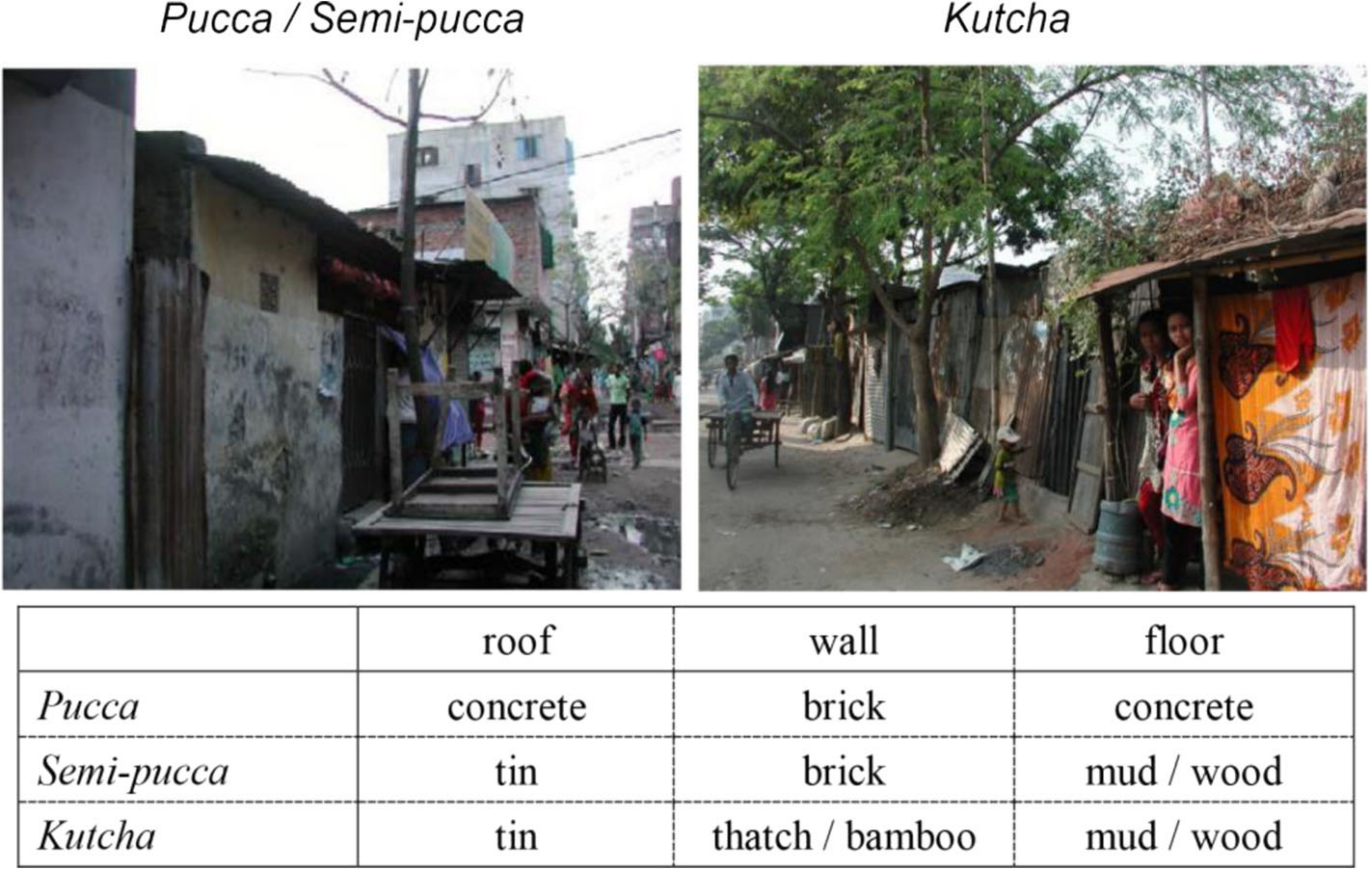
Types of housing structure

Figure 3 shows examples of types of housing structure. About 39% lived in single or multistoried houses with concrete roofs, concrete floors, and brick walls (*pucca*), 30% in houses with tin roofs, mud or wooden floors, and brick walls (*semi-pucca*), and 31% in houses with tin roofs, mud or wooden floors, and walls made of thatch or bamboo (*kutcha*) (Table 1). It was found that about 84% of the population shared toilets, and 81% shared kitchens with other households. Almost all the population used piped water as their source of drinking water, and 62% satisfied with the water quality.

To categorize household wealth levels, we referred to the 2010 STEPS survey’s wealth index constructed from the asset information including the type of main material used for the roof, wall and floor of the main house and household ownership of electricity, flush toilet, television, refrigerator, car, etc. [4]. Adapting the national survey’s asset information to the urban poor community where diversity in wealth might be less than that of the target of the nation-wide survey, we used the type of housing structure as a proxy indicator of the household wealth, and categorized household wealth levels into 2 groups: “lower-middle wealth” households were defined as those living in *pucca*; and “low wealth” households were defined as those living in *semi-pucca* or *kutcha*. Lower-middle wealth households tended to have their own kitchens and toilets, while several low wealth households shared a kitchen and a toilet. We found that 39% of the population of the community were the lower-middle wealth group, while 61% were the low wealth group.

About 52% of adults had at least 5 year education, and 28% had secondary or higher level education. About 26% of adults were employed, 19% were day laborers, and 16% were self-employed.

Table 2 shows self-reported health related indicators of adults aged 18 years and older who responded the interview representing each household (*n* = 7616), by age group and sex. The number of women was much larger than that of men among the respondents, as women were more likely to be at home when interviewers visited, while men tended to be working outside. Tobacco product use was prevalent in men regardless of age and in women aged over 35 years, although they might be underreported. Cigarette smoking was prevalent in men regardless of age, but very few women smoked. Smokeless tobacco chewing was prevalent both in men and women aged over 35 years.

**Table 2:** Valid percentages of self-reported health indicators among respondents aged 18 years or more (*N* = 7616)

Age group	Men							Women							*p* ^a^
	18–24	25–34	35–44	45–54	55–64	65+	All	18–24	25–34	35–44	45–54	55–64	65+	All	
Number	353	587	383	187	89	73	1672	1583	2366	1106	500	249	140	5944	
Smoking cigarettes (%)	33.0	52.2	63.1	52.9	44.9	52.1	50.4	1.0	1.2	1.9	1.4	0.4	1.4	1.3	<0.001
Chewing smokeless tobacco (%)	7.7	17.4	33.2	47.3	48.9	49.3	25.4	5.0	13.9	35.3	53.0	64.1	74.1	22.3	0.305
Tobacco product use (%)	36.1	57.4	75.3	74.9	75.3	79.5	61.0	5.9	14.8	36.6	53.8	64.3	74.1	23.2	<0.001
Hypertension (%)															<0.001
Yes	1.4	3.1	5.2	11.8	22.5	17.8	5.9	2.5	5.5	13.1	21.6	24.9	23.6	8.7	
No	7.4	14.8	17.0	19.3	19.1	21.9	14.8	15.7	20.6	22.1	24.6	18.9	13.6	19.7	
Unknown	91.2	82.1	77.8	69.0	58.4	60.3	79.4	81.9	73.8	64.8	53.8	56.2	62.9	71.6	
Diabetes (%)															<0.001
Yes	0.3	2.0	3.1	11.8	5.6	12.3	3.6	0.3	2.2	8.2	13.6	15.7	13.6	4.6	
No	5.9	8.9	9.9	13.9	21.3	21.9	10.3	8.8	11.9	15.6	16.2	17.7	13.6	12.4	
Unknown	93.8	89.1	86.9	74.3	73.0	65.8	86.1	90.9	85.9	76.1	70.2	66.7	72.9	83.0	
Heart Disease (%)															<0.001
Yes	0.3	1.4	3.9	3.2	9.0	6.8	2.6	1.5	2.6	5.5	9.8	13.7	12.1	4.1	
No	3.7	5.3	4.4	6.4	11.2	12.3	5.5	3.2	4.0	4.9	7.0	5.6	3.6	4.3	
Unknown	96.0	93.4	91.6	90.4	79.8	80.8	91.9	95.3	93.4	89.6	83.2	80.7	84.3	91.6	
Stroke (%)															0.649
Yes	3.4	5.1	2.1	2.7	7.9	6.8	4.0	3.3	4.6	2.1	4.2	3.6	5.0	3.7	
No	0.0	1.2	3.4	5.3	6.7	12.3	2.7	0.0	1.3	4.4	7.0	8.8	7.1	2.5	
Unknown	96.6	93.7	94.5	92.0	85.4	80.8	93.3	96.7	94.1	93.5	88.8	87.6	87.9	93.8	
Diarrhea^b^ (%)	2.9	3.1	3.1	3.7	2.2	4.1	3.1	3.9	4.1	5.2	6.2	6.4	3.6	4.5	0.007

^a^Differences between all men and all women were tested by using age-adjusted binary and multinominal logistic regression models for dichotomous and trichotomous variables, respectively

^b^Suffering from diarrhea in the past 30 days

Prevalence of self-reported hypertension and diabetes were 5.9 and 3.6% in men and 8.7 and 4.6% in women, respectively, both higher in women than in men. Prevalence of self-reported heart disease and stroke were 2.6 and 4.0% in men and 4.1 and 3.7% in women, respectively. Around 70 to 90% of the population responded that they did not know if they had hypertension, diabetes, heart disease, or stroke. A few people in all age groups reported that they had stroke, however, it was likely that they confused all types of fainting incidents as stroke. Incidences of diarrhea in the last one month were about 3% in men and around 5% in women.

## Discussion

This study showed population profile and residential environment of the people living in an urban poor community in Bangladesh. Several demographic registration systems were established in some rural areas in Bangladesh [8], however, complete count surveys in very crowded urban shantytowns were rare. The recent 2011 census, which took several years to complete, might not reflect the profile of often fluctuating urban poor population in details [14].

The population pyramid was partly similar to the constricted and stationary types, but had a large proportion of younger population and a small proportion of elder population. The population composition was different from the expansive type reflecting high fertility and high mortality, or the stationary and constrictive types in high income countries where both low fertility and low mortality were achieved. The population pyramid might reflect declined fertility in the community, while mortality of elder people had not much decreased. The population composition might result from the influx of young population to the urban community from other areas. Population changes caused by migration might be more significant than the natural changes in the urban shantytown [15, 16].

About half of the adults did not complete primary level education and 32% had never received formal education, which was consistent with the adult literacy rate (61% in 2013) reported by the Bangladesh government [17]. However, primary school enrollment of the children in the community is expected to be high, as there are primary schools in the community, in line with the national average (98% primary school enrollment in 2014).

Ratio of employed people was higher than day laborers or self-employed, perhaps because the community located close to garment factories, which hired many manual laborers [13]. Most day laborers involved in hard physical works such as puling cycle rickshaws, and self-employed included street vendors of snacks, tobacco, and daily commodities. Married women tended not to work outside as shown that 28% of adults were homemakers.

We found that households in the urban poor community were not equally poor anymore, although their wealth levels were similar when the community was established 44 years ago. Along with the overall economic development, some of the current residents were relatively well-off by buying up several plots to build brick houses, while others remain very poor sharing shanties made of bamboo and tin. It was difficult to estimate incomes of the people in shantytowns, of whom majority were day laborers, however, we could categorize household wealth levels by using types of housing as a proxy indicator. We found that about 39% of the population was relatively well-off, although they still lived in the shanty area, perhaps because it was convenient to stay in the middle of the city.

Although 84% of the population did not have toilets in their house, most people had access to safe piped water. Each household was provided a tube well by the government when the community established 44 years ago. Then, several environmental improvement programs sponsored by aid agencies had been conducted in the area [11, 12], which included providing piped water and cleaning sewage ditches. Although arsenic contamination of groundwater was widely observed in Bangladesh [18], piped water supply in the community had been confirmed to be free of arsenic contamination. Good access to safe water was supposed to bring the relatively low incidence of diarrhea.

A previous study on urban slums in 6 cities in Bangladesh reported household conditions in Dhaka slums as follows: about 92% used piped water supply, 99% shared toilet, only 12% owned house, 46% lived in *kutcha* and 52% lived in *semi-pucca* [19]. Our findings showed that living conditions of our target area were much better than those of 4966 slum clusters studied in 2005. This may be attributable to significant improvement of living conditions in Dhaka in the past decade along with rapid economic development. This may also indicate the relatively good living condition of our targeted area, which was originally a settlement established by the government.

Another study on health and living conditions in 8 Indian cities reported that household conditions in Kolkata slums as follows: 85% used piped water supply, 75% shared toilet, and only 7% lived in *kutcha* or *semi-pucca* [20]. The study also reported that not all slum dwellers were poor, and proportion of the population of under 15 years of age was less than 30% as fertility declined, while the proportion of 15 to 59 age group was high due to migration. These findings in India were similar to our findings.

High prevalence of tobacco use was confirmed in this study, in consistent with previous studies in Bangladesh [21, 22]. Women chewed tobacco more often than men, but refrained smoking cigarettes. This indicated that chewing tobacco products were culturally tolerated, considering that Bangladesh women observed various cultural norms and behavioral restrictions. Tobacco chewing seemed to be more commonly practiced among people aged over 35 years than young people in the 20s, suggesting that tobacco chewing was a traditional old habit while cigarette smoking was a relatively new habit. Different approaches for men and women, and for young and old, need to be developed to control tobacco.

The observed low prevalence of hypertension compared to the crude estimate by WHO (men 22.0%; women 21.0%) [2] may be resulted from suboptimal awareness of the disease, since majority of respondents in the present study did not know whether they had hypertension or not. Similarly, the prevalence of diabetes was about half of the WHO estimated national prevalence (men 8.6%; women 7.4%) [23]. Furthermore, the accuracy of the self-report is unknown.

The high ratio of the people who did not know they had NCDs or not may be in part due to the health systems unprepared to work on NCD control and management. Health systems had been designed to target infectious diseases and maternal and child health, thus health professionals in the community often lacked proper trainings for managing NCDs. Most of the clients of community health centers were women and children, therefore, adult men tended to use casual health check-ups provided by auxiliary pharmacists or street vendors [24], whose diagnosis of hypertension and diabetes were unlikely to be reliable. Higher prevalence or awareness in women than in men may suggest more frequent use of health services by women than men, *e.g.*, blood pressure measurements during antenatal care. Further studies are required to investigate prevalence of NCD risk factors.

The high prevalence of unawareness may be also due to the lack of opportunities for the people to obtain accurate knowledge of NCDs. Since most NCDs do not show specific symptoms in the beginning, it is very important to provide health education about risk factors and early symptoms of NCDs. The prevalence of unawareness was higher in the young than the old, despite their higher school attendance. Health education for NCD prevention should be incorporated into formal education.

The strength of this study was that we conducted complete count survey targeting an urban shantytown, where households were too crowded to survey accurately and population often fluctuated due to migration. We expect the findings of this study would be utilized as a baseline population profile for the future epidemiological studies in the area. We have already conducted an epidemiological study of NCD risk factors using representative sampling methods based on the baseline data.

The limitation of this study was that we targeted only one urban poor community, excluding surrounding shantytowns, thus the situation might be somewhat different from that of other urban poor communities. Another limitation was that we interviewed only one person in the household, who might have given incorrect information of other household members. The respondent in the household was not chosen through a representative sampling methods, either. Furthermore, all health related information were self-reported based on the past history, but were not clinically confirmed, therefore, current conditions might be underreported.

In conclusion, this study showed population profile and residential environment in an urban poor community by a complete count survey. We expect the study to serve as a baseline for future epidemiological studies.
